# Hyperglycaemia, Insulin Therapy and Critical Penumbral Regions for Prognosis in Acute Stroke: Further Insights from the INSULINFARCT Trial

**DOI:** 10.1371/journal.pone.0120230

**Published:** 2015-03-20

**Authors:** Charlotte Rosso, Christine Pires, Jean-Christophe Corvol, Flore Baronnet, Sophie Crozier, Anne Leger, Sandrine Deltour, Romain Valabregue, Mélika Amor-Sahli, Stéphane Lehéricy, Didier Dormont, Yves Samson

**Affiliations:** 1 Centre de Recherche de l'Institut du Cerveau et de la Moelle épinière, Paris, France; 2 UPMC Paris 6, Inserm, U1127; CNRS, UMR 7225, Paris, France; 3 CONAM, UPMC Paris 6, Inserm, U1127, CNRS, UMR 7225, Paris, France; 4 APHP, Urgences Cérébro-Vasculaires, Hôpital Pitié-Salpêtrière, Paris, France; 5 Institut du Cerveau et de la Moelle épinière, Centre de Neuro-Imagerie de Recherche (CENIR), Paris, France; 6 INSERM, APHP, Centre d’Investigation Clinique CIC9503, Département des Maladies du Système Nerveux, Hôpital Pitié-Salpêtrière, Paris, France; 7 APHP, Service de Neuroradiologie, Hôpital Pitié-Salpêtrière, Paris, France; 8 COGIMAGE, UPMC Paris 6, Inserm, U1127, CNRS, UMR 7225, Paris, France; St Michael's Hospital, University of Toronto, CANADA

## Abstract

**Background:**

Recently, the concept of ‘clinically relevant penumbra’ was defined as an area saved by arterial recanalization and correlated with stroke outcome. This clinically relevant penumbra was located in the subcortical structures, especially the periventricular white matter. Our aims were to confirm this hypothesis, to investigate the impact of admission hyperglycemia and of insulin treatment on the severity of ischemic damages in this area and to study the respective contributions of infarct volume and ischemic damage severity of the clinically relevant penumbra on 3-month outcome.

**Methods:**

We included 99 patients from the INSULINFARCT trial. Voxel-Based Analysis was carried on the Apparent Diffusion Coefficient (ADC) maps obtained at day one to localize the regions, which were more damaged in patients i) with poor clinical outcomes at three months and ii) without arterial recanalization. We determined the intersection of the detected areas, which represents the clinically relevant penumbra and investigated whether hyperglycemic status and insulin regimen affected the severity of ischemic damages in this area. We performed logistic regression to examine the contribution of infarct volume or early ADC decrease in this strategic area on 3-month outcome.

**Findings:**

Lower ADC values were found in the corona radiata in patients with poor prognosis (p< 0.0001) and in those without arterial recanalization (p< 0.0001). The tracking analysis showed that lesions in this area interrupted many important pathways. ADC values in this area were lower in hyperglycemic than in normoglycemic patients (average decrease of 41.6 ± 20.8 x10^−6^mm^2^/s) and unaffected by the insulin regimen (p: 0.10). ADC values in the clinically relevant penumbra, but not infarct volumes, were significant predictors of 3-month outcome.

**Conclusion:**

These results confirm that the deep hemispheric white matter is part of the clinically relevant penumbra and show that hyperglycaemia exacerbates the apparition of irreversible ischemic damage within 24 hours in this area. However, early intensive insulin therapy fails to protect this area from infarction.

**Trial Registration:**

ClinicalTrials.gov NCT00472381

## Introduction

The localization of the clinically relevant penumbra region could be essential to decision making in acute stroke treatment (such as thrombolysis) and to estimate stroke outcome. [1]. Recent Magnetic Resonance Imaging (MRI) image-processing studies of acute Middle Cerebral Artery (MCA) infarct have shown that the clinically relevant penumbra i.e., the area that is saved by arterial recanalization *and* associated with poor outcome [2, 3], was located in subcortical areas such as the periventricular white matter (see review in [4]). In line with these findings, mathematical models suggest also that disruption of long-range connections may be especially harmful for functional recovery in large brains [5]. Moreover, other studies suggest that ischemic damage in this clinically relevant penumbra area is more important for outcome than the infarct volume [6, 7]. Here, we attempted to replicate our findings [3,4] in INSULINFARCT (Intensive versus Subcutaneous Insulin in Patients with Hyperacute Cerebral Infarction) patients [8] (NCT00472381, URL: http://clinicaltrials.gov). This substudy was a non pre-specified post-hoc analysis. In addition, because admission hyperglycemia has been consistently correlated with poor outcomes [9–11] and a reduced amount of salvaged tissue in the ischemic penumbra at the acute phase [12–17], we investigated whether hyperglycemic status at admission correlated with more severe ischemic damage in the clinically relevant penumbra area. In the INSULINFARCT trial, we randomly divided 180 patients with < 6 hours carotid territory stroke into groups receiving intensive insulin therapy or standard subcutaneous insulin treatment [8]. Although intensive insulin therapy significantly improved glucose level control, no effect on clinical outcome was detected, as has also been reported in a recent meta-analysis [18] and in other studies [19, 20]. Furthermore, intensive insulin therapy was paradoxically associated with a larger infarct growth [8, 20].

We used whole brain voxel-based analyses of the apparent diffusion coefficient (ADC) maps obtained 24 h after stroke onset. At this time, the degree of ADC decrease reliably predicted the severity of irreversible ischemic damage [21–23]. Voxel-based analyses allowed to detect—without any anatomical *a-priori*—whether given brain areas had statistically lower regional ADC values in one sub-group of patients compared to another [3, 24]. In line with to the previous results, we hypothesized that patients with poor 3-month outcomes and patients without MCA recanalization would have more severe ischemic damages (assessed by ADC values) in the peri-ventricular white matter, in nearly co-localized areas. We also predicted that hyperglycemic status, but not insulin treatment regimen given in the first 24 hours, would be associated with lower ADC values in the clinically relevant penumbra area. Finally, we expected that the severity of the ischemic damage in this region, which might interrupt many long-range connections, would be more important than the infarct volume for 3-month clinical outcomes.

## Methods and Materials

### Population

**Fig 1:**
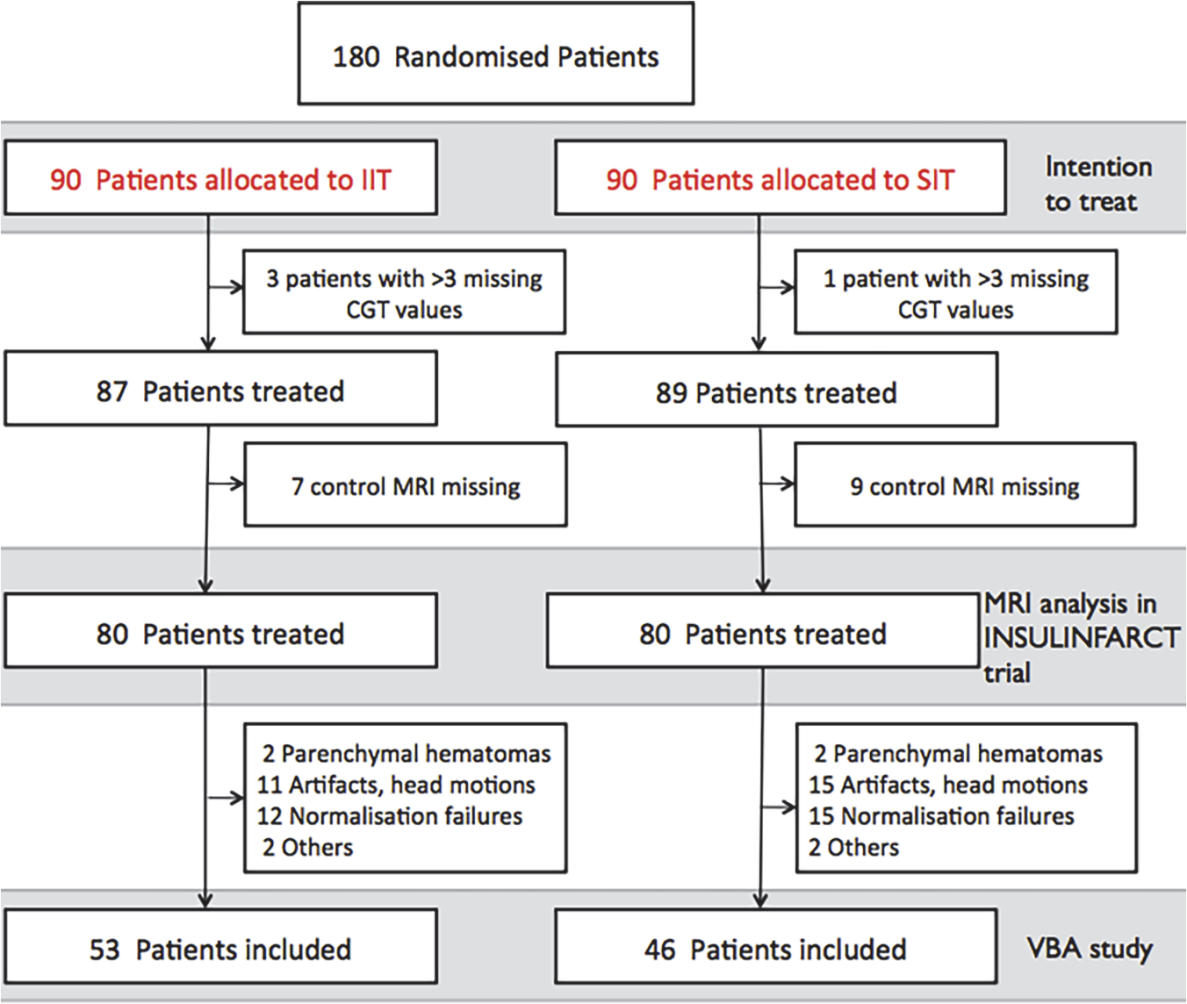
Flow chart of the study. CGT means Capillary glucose test. As pre-specified in the main protocol, patients for whom ore than 3 CGTs were missing during the treatment had to be excluded. VBA means voxel-based analysis and the VBA study refers to the study presented in this paper.

In the INSULINFARCT trial (NCT00472381, URL: http://clinicaltrials.gov) [8], 180 patients with MRI-demonstrated ischemic stroke and NIHSS from 5 to 25 at admission (< 6 hours) were randomized between June 2007 and March 2011 to receive intensive intravenous insulin or usual subcutaneous insulin for 24 hours. Day one MRI scans was performed to monitor infarct growth and arterial recanalization. The 3-month clinical follow-up using the modified Rankin scale (mRS) was completed. The protocol for this trial and supporting CONSORT checklist are available as supporting information (see S1 Checklist and S1 Protocol). In the intention-to-treat analysis, the intensive insulin regimen improved glucose control during the first 24 hours but did not modify the 3-month functional outcome, and it was associated with unexpectedly larger infarct growths although insulin treatment was initiated within 6 hours post-stroke onset. The MRI infarct volume analysis was performed in 160 patients, but 61 of these patients had to be excluded from the present analysis due to technical problems related to preprocessing (brain spatial normalization steps; see below and flow chart in Fig. 1). Therefore, 99 patients were included in the present study. They were divided into groups as follows: i) good outcome group (GO, defined as independency by a 90-day modified Rankin Scale from 0 to 2, n = 54) *vs*. poor outcome (PO, defined as moderate to severe disability with mRS 3 to 5, n = 43) and (ii) patients with complete or partial MCA recanalization at 24 hours (n = 77) *vs*. patients without MCA recanalization (n = 20). Patients with complete or partial recanalization were considered as “recanalized” patients because partially recanalized patients had a more favorable course than patients with persistent occlusion in previous studies [16, 25]. Partial recanalization was defined as at least minimal flow related enhancement in the region of a previously identified MCA clot or displacement of the initial clot to a more distal portion of the MCA.

Two patients had missing data on the modified Rankin scale because they were lost at the 3-months follow-up and two other patients had no interpretable magnetic resonance angiography at day one to characterize the recanalization status.

The INSULINFARCT study was conducted according to established clinical practice guidelines and was approved by the local ethical committee (Paris VI IRB). Written informed consent from each participant or from a legal proxy/family member was obtained.

### Magnetic Resonance Imaging

#### Acquisition

The initial MRI was performed at admission (<5 h), before any randomization, and the control MRI was performed between days one and three. MR imaging was performed on a 3.0 Tesla whole-body MR unit (Signa 3.0-T HDx, General Electric Medical System, Milwaukee) with an 8-channel head coil. The MRI protocol included four sequences: diffusion-weighted imaging (DWI), fluid attenuated inversion recovery (FLAIR), intra-cranial time-of-flight MR angiography and T2*-weighted sequence. Parameters can be found in the S1 File.

#### ADC preprocessing

Quantitative ADC maps were generated using commercial software (Functool 2, General Electric) and spatially normalized to the Montreal Neurological Institute (MNI) reference frame using the T2-weighted template from SPM8 (Wellcome Department of Cognitive Neurology, London, UK, http://www.fil.ion.ucl.ac.uk/spm/). Each normalized ADC map was visually checked as reported in [26] for misregistration. The spatial normalization of MRI images from acute stroke patients obtained in clinical routine remains a technically challenging step, one which resulted in the exclusion of 26 patients because of artifacts (ghosting, head motions or large susceptibility artifacts) and of 27 patients because of normalization failures (mainly explained by the lack of slices at the top of the head). The successfully spatially normalized ADC maps with infarct lesions located in the left hemisphere (n = 48) were flipped to the right. Therefore, all infarcts were located in the same hemisphere. ADC maps were then smoothed by a 4 mm Gaussian filter in each direction (x, y, and z), as performed in one of our previous studies [3].

### Statistical analysis

#### Descriptive statistics

Descriptive statistics included the median and the interquartile range (25^th^ and 75^th^ percentiles, IQR). Kolmogorov-Smirnov test was used to confirm that the data had a normal distribution. Then, comparisons between patients’ baseline characteristics were made using independent sample t-tests with unequal variance when appropriate. All descriptive statistical analyses were performed using the SPSS software (version 20).

#### Voxel-based analysis

We performed two voxel-based analyses using SPM8 with the smoothed and normalized ADC maps (acquired on the day one MRI) by running two-sample-tests: Good *vs*. Poor outcome patients and Recanalized *vs*. Non-Recanalized MCA patients. In these general linear models, age was entered as an adjusting covariate because it could influence the ADC values. The analysis was conducted using a height threshold of 0.001 (uncorrected) and corrected at the cluster level by a Family Wise Error (FWE) threshold at p<0.01 for multiple comparisons. We compared directly the PO *vs*. GO group and the recanalized *vs*. non-recanalized groups at day one in order to detect age-corrected significant areas of ADC decrease occurring selectively in the poor (*vs*. good) outcome and the non-recanalized (vs. recanalized) groups. The three most significant peaks for each contrast were described in terms of location and T-values using the Jülich Atlas [27].

Then, to better characterize the areas where poor outcome-related ischemic damage co-localized with areas associated with non-recanalization-related ischemic damage, we computed the intersection of the two clusters using MRIcron (http://www.cabiatl.com/mricro/mricron). As we noted in the introduction, this intersection corresponded to the clinically relevant penumbra. We performed a fiber tracking analysis (in a subject from the connectome PROJECT, www.humanconnectomeproject.org/) in order to identify the fiber tracts likely to be interrupted by lesion of this area. Details on fiber tracking and diffusion tensor processing are given in the S2 File.

#### Impact of hyperglycemic status and insulin regimen on the severity of damages to the clinically relevant penumbra

We then extracted the individual ADC values in this area and in the mirror area in order to obtain absolute (ADC_abs_ and ADC_cl_) and ratio values (ADC_r_: ADC_abs_/ADC_cl_*100). In order to investigate the potential effects of hyperglycemic status (defined as a capillary glucose test equal or up to 7 mmol/l as in the main protocol) and insulin treatment regimen on the severity of ischemic damage in this area, we performed multiple regression analyses using ADC_abs_ as the dependent variable and hyperglycemic status, insulin regimen, age, ADC_cl_, MRI delay since stroke onset, MCA recanalization, and infarct volume as independent variables. The same analysis was conducted on the ADC_r,_ except that ADC_cl_ was not included as a variable.

#### Respective contributions of infarct volume and severity of ischemic damage of the clinically relevant area for the functional outcomes

We performed logistic regression in order to investigate whether the severity of ischemic damage in the clinically relevant penumbra area or the infarct volume or both was an independent predictor of good outcomes (mRs 0–2) or very poor outcomes (mRs 4–6). Age was also entered as a covariate because it could influence both ADC values and functional outcomes.

## Results

### Patients

The 99 patients included tended to have lower 24 h-mean capillary glucose tests (p: 0.045) and lower day one infarct volumes (p: 0.03) than those who had to be excluded for image artifact or normalization failures, but they had similar other baseline characteristics (S1 Table). The characteristics of the patients and of the subgroups are given in Table 1. As expected, patients with good outcome were younger (p: 0.01), were less severe than patients with poor outcome (p<0.0001 in their initial and day seven NIHSS), and had lower baseline glycemic levels (p<0.0001) and tighter glucose control (p: 0.0008, even if the type of insulin regimen were in the same proportion in the two groups). Despite the same MRI delay, they had smaller infarct volumes at admission (p: 0.02) and day one (p<0.0001), and they tended to have higher proportions of recanalization (p: 0.1).

**Table 1:** Characteristics of the patients.

Median, IQR	All	Good outcome	Poor Outcome	Recanalized	Non-recanalized
	N = 99	N = 54	N = 43	N = 77	N = 20
**Clinical data**					
Age (years)	70 56–82	63.9*	78	73.2	70.8
	56–82	54–78	57–85	56–83	52–81
Gender (n,%) male	57	35/54	21/43	48/77	9/20
	57%	64%	49%	62%	45%
Initial NIHSS	13	11*	19	13	15
	8–19	7–14	12–22	8–19	11–19
Day 1 NIHSS	9	4*	16	9**	15
	3–17	2–8	11–19	3–15	7–19
3-month mRS 0–2 (n, %)	54/97	54	0	31/75	12/20
	56%	100%	0%	41%	60%
**Glycaemic data**					
Initial CGT (mmol/l)	6.7	6.2*	7.4	6.8	6.7
	5.8–8.1	5.4–7.1	6.4–8.4	5.8–8.1	6–8
Mean 24-h CGT (mmol/l)	5.8	5.6*	6.1	5.8	5.9
	5.3–6.6	5.1–6.3	5.5–7	5.2–6.6	5.3–6.6
Insulin regimen (SIT) n,%	46	26/54	18/43	35/77	9/20
	46%	48%	42%	45%	45%
**MRI data**					
Time to FU MRI (hours)	29.7	30	30.2	30.8	27
	26.3–36.1	26.4–35.7	26.5–39.8	27.7–36.5	25.3–31.2
Recanalization (n, %)	77/9	44/52	31/43	77	0
	79%	85%	71%	100%	0%
Initial Volume (cm^3^)	8.8	4.8*	12.6	8.5	11.4
	2.6–19	0.9–17.1	6.8–32.3	2.2–24.4	3.5–33.3
Follow-up volume (cm^3^)	23.9	14.3*	54.9	22.5**	63.1
	5.8–72	2–31.2	18.9–144.6	4.8–55.8	6.2–155
ADC_abs (x 10_^–6^_mm_^2^_/s)_	730	776*	656	756**	585
	598–807	678–824	488–782	658–819	414–713
ADC_cl (x 10_^–6^_mm_^2^_/s)_	796	791	802	805	786
	763–858	752–856	779–859	770–862	734–813
ADC_ratio_	0.90	0.94*	0.79	0.92**	0.74
	0.78–0.97	0.89–1	0.64–0.91	0.81–0.97	0.50–0.87

* p< 0.05 for the comparison between poor and good clinical outcome;

** p<0.05 for the comparison between recanalized and non-recanalized patients: mRS: modified Rankin scale: CGT: Capillary Glucose Test, SIT: Subcutaneous insulin therapy: FU: Follow-up.

In the comparison between recanalized and non-recanalized patients, they had similar characteristics at baseline. At follow-up, recanalized patients had smaller follow-up infarct volume (p: 0.013) and less severe NIHSS at days one and seven (p: 0.03 for both) than non-recanalized patients.

### Voxel-based analyses: poor outcome-related and MCA recanalization-related areas

**Fig 2:**
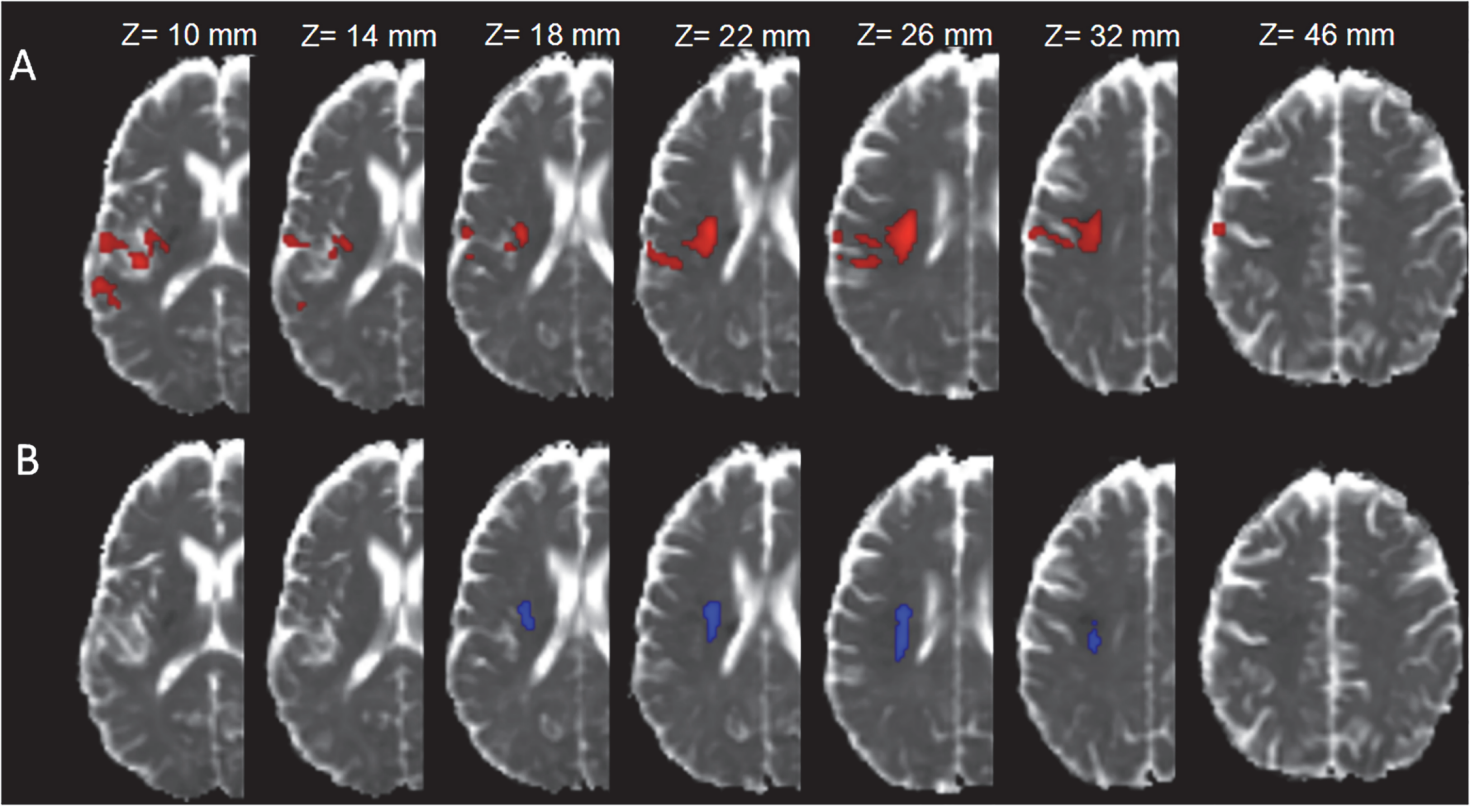
Region with lower ADC values at day one in non-recanalized patients and in poor outcome patients. A. Superposition of the area of day-one ADC decrease associated with 90-day poor outcome (in red) on ADC images (MNI space, z-coordinates). B. Superposition of the area of day-one ADC decrease associated with non-recanalized (vs. recanalized) patients (in blue) on ADC images (MNI space, z-coordinates). Images are shown using neuroradiological conventions (right hemisphere on the left side).

**Fig 3:**
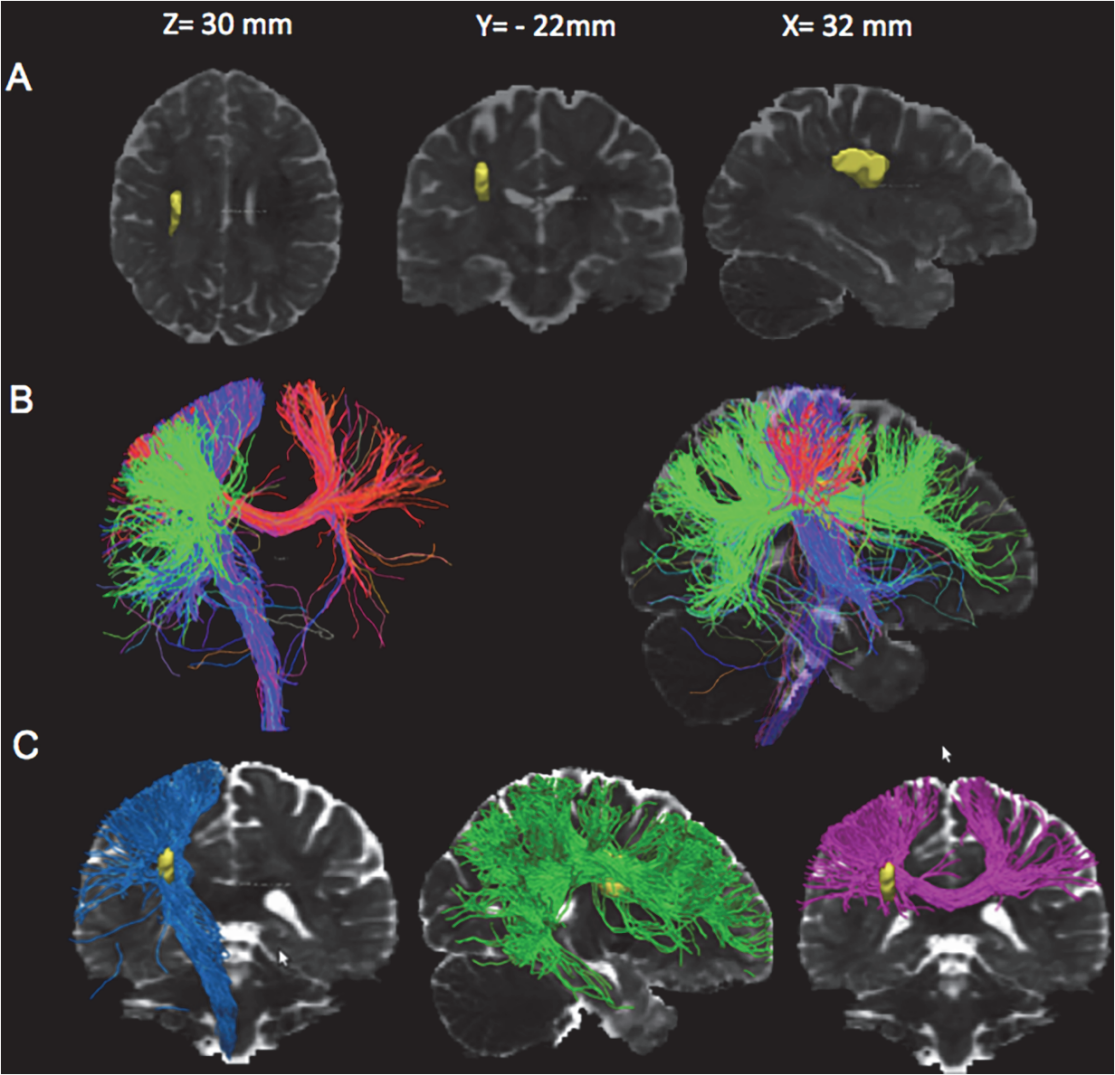
Tracking analysis from the strategic area. Color code is directional (blue for z-axis, green for y-axis and red for x-axis). A. Superposition of the strategic area (in yellow) on a normalized ADC map. B Association and Projection tracts passing through the strategic area on a coronal view (left image) and on a sagittal view of a normalized ADC map. The color code is directional (pink for the x-axis, blue for z-axis and pink for y-axis). C. Association and Projection tracts travelling though the strategic area (in yellow). Left: The projections tract projections at the top in the following areas: primary motor, premotor, and parietal cortices (blue); Middle: The association tract passing through the yellow region contains part of the superior longitudinal fasciculus (green); Right: The fibers of the corpus callosum passing through the strategic area (pink).

The comparisons of the good *vs*. poor outcome patients showed that poor outcome patients had significantly lower ADC values, indicating more severe ischemic damage in a single cluster of voxels (p < 0.001, 10 ml) localized in the peri-ventricular white matter and the adjacent parieto-temporal cortex (Fig. 2A). The comparison of patients with and without MCA recanalization showed that non-recanalized patients had significantly lower ADC values in a smaller cluster of voxels (1.8 ml, Fig. 2B) also located in the periventricular white matter. The peak coordinate locations are reported in Table 2. The overlap between both regions (an operational definition of the clinically relevant penumbra, since this area is spared in patients with arterial recanalization and good functional outcome) had a volume of 1.2 ml, corresponding to 12% of the poor outcome-related and 67% of the non-recanalized ischemic area. In this overlap region (Table 1), the ADC_abs_ values and the ADCr were lower (p < 0.0001) in the poor outcome group than in the good outcome group and in the non-recanalized *vs*. recanalized patients (p<0.0001). Compared to the ADC values in the mirror region, these values corresponded to a 21% decrease in the poor outcome group, a 6% decrease in the good outcome group, a 26% decrease in the non-recanalized and an 8% decrease in the recanalized group. Our tracking analysis showed that the overlap area is crossed by projection fibers including the cortico-spinal tract, inter-hemispheric callosal fibers at the central sulcus level, and a part of the intra-hemispheric superior longitudinal fasciculus (Fig. 3).

**Table 2:** Peak coordinates (x, y, z in mm in the MNI space) and localization of the clusters related to the difference between the groups of patients.

Contrasts	Location	T	x	y	z
NR. vs. R	226 voxels				
	**Corona Radiata_CST**	4.2	28	−10	24
	**Corona Radiata_SLF**	3.6	28	-22	26
GO vs. PO	1328 voxels				
	**Periventricular white matter_CST**	5	28	−16	24
	**Temporoparietal junction**	4.8	46	−28	0
	**Temporoparietal junction**	4.7	54	−36	6

T is the value of the T-statistic. NR denotes the non recanalized patients and R the recanalized patients; GO: Good outcome defined as a modified Rankin Scale from 0 to 2; PO: Poor outcome defined as a modified Rankin Scale from 3 to 6; CST denotes corticospinal tract and SLF superior longitudinal fasciculus

### Impact of hyperglycemic status and insulin regimen on the severity of damages to the clinically relevant penumbra

The multiple regression model explained 71% in the variance of the decrease in ADC_abs_ in the clinically relevant penumbra region. The significant parameters were ADC_cl_ values, age (with an average decrease in ADC_abs_ of 19 ± 6.9 x10^–6^mm^2^/s per decade), hyperglycemic status (with an average decrease in ADC_abs_ of 41.6 ± 20.8 x10^–6^mm^2^/s), persistent occlusion of MCA (with an average decrease in ADC_abs_ of 77.9 ± 23.8 x10^–6^mm^2^/s), and infarct volume (with an average decrease in ADC_abs_ of 12.5 ± 1.3 x10^–6^mm^2^/s per 10 ml). The post-stroke delay of MRI had a non-significant effect on ADC_abs_ values (p = 0.10), and the insulin regimen had no detectable effect (p = 0.70).

For the ADC ratio, the multiple regression model explained 62% of the variance. The significant parameters were age (with an average decrease of 1.9 ± 0.8% per decade), hyperglycemic status (average decrease of 5.3 ± 2.6%), persistent occlusion of MCA (average decrease of 8.9 ± 2.9%) and infarct volume (average decrease of 1.5 ± 0.2% per 10 ml). Post-stroke delay of MRI (p = 0.11) and insulin regimen (p = 0.90) were not significant.

As this type of analysis do not test specifically an interaction between recanalisation and glycemic status on ADC values, we performed additionally an ANCOVA analysis with the ADC_r_ as the dependent variable, the MCA recanalisation, and the glycemic status as the between subject factors. There was a main effect of the glycemic status (F(3,89): 4.1, p: 0.04) and a main effect of MCA recanalisation (F(3,89): 15.8, p<0.001) but not a significant interaction (p: 0.25) suggesting that the effect of glycemic status was present in recanalized and non-recanalized patients (S1 Fig.).

### Respective contribution of infarct volume and severity of ischemic damages of the clinically relevant area to the functional outcome

The logistic regression model predicting good outcome showed that the significant predictors were age (OR per decade: 0.58; 95% CI: 0.40–0.83, p: 0.003), ADC_cl_ (OR per 10 units: 0.91; 95% CI: 0.84–0.99, p: 0.02), and ADC_abs_ in the clinically relevant penumbra region (OR per 10 units: 1.10; 95% CI: 1.03–1.16, p: 0.005). The effect of infarct volume was not significant (OR per 10 ml: 0.94; 95% CI 0.83–1.06, p: 0.30). The model correctly classified 77% of the patients.

The logistic regression model predicting very poor outcome (mRS 4 to 6) showed again that the significant predictors were age (OR per decade: 2.39; 95% CI: 1.46–3.92, p: 0.0005), ADC_cl_ (OR per 10 units 1.10; 95% CI 1.007–1.21, p: 0.04), and ADC_abs_ (OR per 10 units: 0.89; 95% CI: 0.84–0.95, p: 0.001), but not infarct volume (OR per 10 ml: 1.02; 95% CI: 0.91–1.14, p: 0.78). The model correctly classified 84% of the patients. Very similar results were found when the logistic regressions were run using ADC_r_ rather than absolute values. ADCr and age were retained in the model, but not infarct volume, with 86% of the patients correctly classified.

## Discussion

In a recent review, we proposed that the clinically relevant penumbra in carotid territory infarcts may be preferentially located in the periventricular white matter and the adjacent internal capsule because the severity of ischemic damage in this area at day one correlated with clinical outcome and because this area appears to be salvaged by early recanalization [3]. The present results are in agreement with this hypothesis because the voxel-based analysis identified a region centered on the superior corona radiata that had lower ADC values at day one post-stroke (indicating more severe ischemic damages) in the 3-month poor outcome *vs*. good outcome comparison. A smaller but overlapping region also had lower ADC values in patients without arterial recanalization. The intersection of both regions, which may represent the clinically relevant penumbra, was very close in location compared to the one described in [3]. One difference in the location was that the one reported in [3] also covered the lenticular nucleus and part of the internal capsule. In the present study, the clinically relevant penumbra region was a purely white matter area centered on the corona radiata.

The corona radiata is a crossroads between projection tracts, transcallosal tracts and association tracts. It seems likely that this region is associated with poor outcome because it creates some type of multiple disconnection syndromes in several main fasciculi. An association between damage to the corona radiata and the modified Rankin scale has also been found in a VLSM study that used Penalized Logistic regression techniques [28]. Among the possible tract disconnections in this study, we could identify easily the corticospinal tract. The corticospinal tract is the main output for motor control and has been already reported as a major determinant of stroke outcome, even at the acute phase [29, 30]. Among the other tracts, the superior longitudinal fasciculus also travels through this region. The superior longitudinal fasciculus is a complex structure [31, 32] that has been divided into multiple parts. The part that is recognizable here is most likely in section III, which is situated in the white matter of the parietal and frontal opercula and extends from the supramarginal gyrus to the ventral premotor and prefrontal regions [33]. This section has also been identified as a component of the arcuate fasciculus, which is involved in language. Superior longitudinal fasciculus lesions could also cause apraxia and neglect because it is a substrate of higher cognitive functions, which could affect stroke outcome [34].

On the other hand, the poor prognosis region is much more extended than the critically relevant penumbra region. It extended more laterally into the corona radiata and involved a significant portion of different cortices (temporoparietal junction, and frontal areas). This area is close to the one we have previously reported (for the subcortical part), but it involved more cortical regions. The hypotheses that could explain this discrepancy are different sample sizes, with higher sample sizes here, which make it possible to detect more differences, and differences in the MRI equipment.

### Impact of HG and insulin regimen on the severity of ischemic damage in the clinically relevant penumbra area

The intersection between the area related to MCA recanalization and the area related to functional outcome corresponds to the clinically relevant penumbra area, for which ischemic damage severity (assessed by the ADC values) is determinant for prognosis. Our multiple regression analysis revealed that baseline hyperglycemia was a determinant factor in the severity of ischemic damage in this area. Hyperglycemia at admission was associated, as already reported by others [9–11], with higher clinical severity and tended to be associated with larger infarct growth. Here, we demonstrated that hyperglycemia worsened the ischemic damage in a strategic region, which was related to functional outcome. This relationship did not exist at admission (<6 hours post-stroke onset), meaning that hyperglycemia most likely reduced the time of salvageable tissue survival.

Finally, there was an interesting “negative” finding in our results. There was no significant effect of the type of insulin regimen on the severity of the ischemic damage in the strategic area, meaning that the intensive insulin regime did not protect against irreversible damages in this area. It was surprising in the main INSULINFARCT study to find that intensive insulin regimen could increase infarct growth, despite better glucose control, even if it was previously reported [20] (for further discussions, see [8]). However, even with increasing volumes, we found no differences in prognosis. In light of these results and the new Cochrane database literature review, we hypothesized that intensive insulin regimen is not efficient because it does not “save” critically relevant regions from damage in the first hours of stroke onset despite a better glucose control. The possible mechanisms of this finding are unknown. One could argue that the glucose control was not achieved fast enough. But the data did not really support this hypothesis since the median delay between stroke and treatment was 174 minutes and 86% of the patients in the intensive insulin regimen had a glycemic value under the target (<7 mmol/l) four hours after treatment initiation. Another explanation may be that the increase in volume may have occurred in heterogeneous locations, too diffuse to be significant.

### Respective contribution of infarct volume and severity of ischemic damage to the clinically relevant area to the functional outcome

We confirmed that 3-month functional outcome is more dependent on the severity of damage to a specific region than the total infarct volume. This finding supports the concept of “localizationist” neurology, in which the site of the damage is more important than the size. This factor is definitely related to the variable eloquence of different brain areas, which forms the basis of the clinico-anatomical approach. The predictive value of infarct volume on stroke outcome has been established [35], but the proportion of the variance of the functional outcome explained by it remains poor [36, 37]. In the Virtual International Stroke Trials Archive (VISTA) database, the infarct volume was an independent predictor of the outcome. However, the volume explained only 15% of the variance [38].

In contrast, in one of our previous studies [6], our multivariate analysis showed that the best predictor of outcome was the degree of ischemic damage (as measured by ADC values at day one) in the ipsilesional corticospinal tract, which classified the patients with 78% accuracy. The infarct volume reached only 69% accuracy. These results and the one reported here emphasize the role of subcortical damage, especially in the corona radiata, in residual disability. This could be easily explained by the fact that this area is at the crossroads of many important pathways in the brain.

Our study has some limitations. Primarily, from the eligible sample size, we lost patients due to the preprocessing of the data (normalization). This reflects the difficulty in properly using clinical routine images as normalized images. However, the excluded patients had similar characteristics to the included ones, limiting the possible bias in comparison to the whole sample size. Second, for the same reason, we flipped left-sided infarcts to the right side. This caused a limitation for the tracking analysis in interpreting the role of the association fibers. For example, the superior longitudinal fasciculus that was identified in the tracking analysis could not have the same importance in left and in the right side strokes.

In conclusion, we confirmed that patients with poor 3-month outcomes and patients without MCA recanalization had more severe ischemic damage at day one in the peri-ventricular white matter, in nearly co-localized areas. We also demonstrated that baseline hyperglycemia might exacerbate the severity of ischemic damage in this area. Intensive insulin therapy failed to save this region in our trial. Finally, we found that the severity of the ischemic damage in the corona radiata, which interrupted many long-range connections, is more important than the infarct volume for 3-month clinical outcome. These results suggest that the efficacy of future drugs that target glucose lowering may be related to their rescue of this deep, clinically relevant area of tissue.

## Supporting Information

S1 Checklist (DOC)

S1 Protocol (PDF)

S1 File MRI parameters. (DOC)

S2 File Diffusion Magnetic Resonance Imaging (dMRI) Analysis. (DOC)

S1 Table Characteristics of the intention-to-treat population in the INSULINFARCT trial, of the included and excluded population for this study. (DOC)

S1 Fig Mean Apparent Diffusion Coefficient values in the clinically relevant penumbra in hyperglycaemic and normoglycaemic patients. Dark grey represents recanalized patients and white bars non-recanalized patients. Bars represent mean; error bars represent 95% confidence interval of the mean.
(TIF)
