# Supplementary material for: Hyperglycaemia, Insulin Therapy and Critical Penumbral Regions for Prognosis in Acute Stroke: Further Insights from the INSULINFARCT Trial
Source: PLoS One. 2015 Mar 20;10(3):e0120230. doi: 10.1371/journal.pone.0120230 (PMC4368038; doi:10.1371/journal.pone.0120230)
Supplement: S1 Protocol — (PDF) [file pone.0120230.s002.pdf]

Stroke
Journal of the American Heart Association

American Heart Association | American Stroke Association

Intensive Versus Subcutaneous Insulin in Patients With Hyperacute Stroke : Results From the Randomized INSULINFARCT Trial

Charlotte Rosso, Jean-Christophe Corvol, Christine Pires, Sophie Crozier, Yohan Attal, Sophie Jacqueminet, Sandrine Deltour, Gurkan Multlu, Anne Leger, Isabelle Meresse, Christine Payan, Didier Dormont and Yves Samson

Stroke. 2012;43:2343-2349; originally published online June 14, 2012;
doi: 10.1161/STROKEAHA.112.657122
Stroke is published by the American Heart Association, 7272 Greenville Avenue, Dallas, TX 75231
Copyright © 2012 American Heart Association, Inc. All rights reserved.
Print ISSN: 0039-2499. Online ISSN: 1524-4628

The online version of this article, along with updated information and services, is located on the World Wide Web at:
http://stroke.ahajournals.org/content/43/9/2343

Data Supplement (unedited) at:
http://stroke.ahajournals.org/content/suppl/2012/06/15/STROKEAHA.112.657122.DC1.html

Permissions: Requests for permissions to reproduce figures, tables, or portions of articles originally published in *Stroke* can be obtained via RightsLink, a service of the Copyright Clearance Center, not the Editorial Office. Once the online version of the published article for which permission is being requested is located, click Request Permissions in the middle column of the Web page under Services. Further information about this process is available in the Permissions and Rights Question and Answer document.

Reprints: Information about reprints can be found online at:
http://www.lww.com/reprints

Subscriptions: Information about subscribing to *Stroke* is online at:
http://stroke.ahajournals.org//subscriptions/

Downloaded from http://stroke.ahajournals.org/ by guest on March 18, 2013

Intensive Versus Subcutaneous Insulin in Patients With Hyperacute Stroke

Results From the Randomized INSULINFARCT Trial

Charlotte Rosso, MD, PhD; Jean-Christophe Corvol, MD, PhD; Christine Pires; Sophie Crozier, MD; Yohan Attal, PhD; Sophie Jacqueminet, MD; Sandrine Deltour, MD; Gurkan Multlu, MD; Anne Leger, MD; Isabelle Meresse, MD; Christine Payan, MD; Didier Dormont, MD; Yves Samson, MD

Background and Purpose—Intensive insulin therapy (IIT) has not yet proven its efficacy on stroke prognosis or in the reduction of MRI infarct growth. The INSULINFARCT study aims at determining in patients with hyperacute stroke whether IIT, with a better control of poststroke hyperglycemia, would reduce subsequent MRI infarct growth than usual care with subcutaneous insulin.

Methods—One hundred eighty patients with MRI-proven ischemic stroke and with National Institutes of Health Stroke Scale from 5 to 25 at admission (<6 hours) were randomized to receive IIT or usual subcutaneous insulin for 24 hours. Admission hyperglycemia was not required for recruitment. Control MRI and 3-month follow-up (with functional outcome and serious adverse events) were planned. The primary objective was to detect a difference in the proportion of patients with mean capillary glucose test <7 mmol/L during 24 hours. The secondary objective was to investigate whether IIT would reduce infarct growth. The analysis was planned in intention-to-treat. Patients with >3 missing capillary glucose test were excluded (n=4).

Results—The proportion of patients with mean capillary glucose test <7 mmol/L in the first 24 hours was higher in the IIT group (95.4% [83 of 87] versus 67.4% [60 of 89]; $P<0.0001$). The infarct growth was lower in the subcutaneous insulin group (median, 10.8 cm^3; 95% CI, 6.5–22.4 versus 27.9 cm^3; 14.6–40.7; 60% of increase; $P=0.04$). The 3-month functional outcome (45.6% [41 of 90] versus 45.6% [41 of 90]), death (15.6% [14 of 90] versus 10% [9 of 90]), and serious adverse events (38.9% [35 of 90] versus 35.6% [32 of 90]) were similar in the subcutaneous insulin and IIT group.

Conclusion—The IIT regimen improved glucose control in the first 24 hours of stroke but was associated with larger infarct growths. IIT cannot be recommended in hyperacute ischemic stroke.

Clinical Trial Registration—URL: http://clinicaltrials.gov. Unique Identifier: NCT00472381.

(*Stroke*. 2012;43:2343-2349.)

Key Words: acute stroke ■ clinical trials ■ hyperglycemia ■ magnetic resonance imaging

Poststroke hyperglycemia is an independent predictor of poor functional outcome and death in the acute phase of stroke[1–4] but this statistical relationship does not prove causality. Indeed, there is still controversy about whether stroke-related hyperglycemia is a cause or effect of the more severe damage found in patients with stroke with elevated blood sugars. However, the hyperglycemia "toxicity" has been suggested by animal studies that reported accelerated penumbra-into-infarction conversion and no-reflow phenomenon.[5] Recent MRI and transcranial Doppler studies have suggested that a similar phenomenon may occur in humans.[6–10] In these studies, evidence has accumulated to define that the glucose toxicity threshold was low (approximately 7 mmol/L, between 6 and 8 mmol/L).[11] These data

Received March 13, 2012; final revision received April 21, 2012; accepted May 8, 2012.

From the AP-HP, Urgences Cérébro-Vasculaires, Pitié-Salpêtrière Hospital, Paris, France (C.R., C. Pires, S.C., S.D., G.M., A.L., I.M., Y.S.); UPMC, Univ Paris 06, Paris, France (C.R., J.-C.C., Y.A., D.D., Y.S.); COGIMAGE, Centre de Recherche de l'Institut du Cerveau et de la Moelle épinière, UPMC Paris 6, Inserm, U975, CNRS, UMR 7225, Paris, France (C.R., Y.A., D.D., Y.S.); AP-HP, Service de Neurologie Pharmacologie Clinique, Pitié-Salpêtrière Hospital, Paris, France (J.-C.C., C. Payan); Centre d'investigation Clinique, INSERM CIC-9503, Pitié-Salpêtrière Hospital, Paris, France (J.-C.C.); Centre de Recherche de l'Institut du Cerveau et de la Moelle épinière, UPMC Paris 6, Inserm, U975, CNRS, UMR 7225, Paris, France (J.-C.C.); AP-HP, Department of Endocrinology, Pitié-Salpêtrière Hospital, Paris, France (S.J.); and AP-HP, Department of Neuroradiology, Pitié-Salpêtrière Hospital, Paris, France (D.D.).

The online-only Data Supplement is available with this article at http://stroke.ahajournals.org/lookup/suppl/doi:10.1161/STROKEAHA.111.657122/-/DC1.

Correspondence to Charlotte Rosso, MD, PhD, Urgences Cérébro-Vasculaires, 47-83 Boulevard de l'Hôpital 75013 Paris, France. E-mail charlotte.rosso@gmail.com

© 2012 American Heart Association, Inc.

***Stroke* is available at http://stroke.ahajournals.org** **DOI: 10.1161/STROKEAHA.112.657122**

Downloaded from http://stroke.ahajournals.org/ by guest on March 18, 2013

explain why hyperglycemia is increasingly considered to be a potential therapeutic target in acute stroke and is of growing interest for intensive insulin treatment (IIT).[11,12]

Currently published randomized trials do not conclude on the clinical efficacy of IIT in patients with stroke.[13–20] The UK Glucose Insulin in Stroke Trial (GIST),[14] which has enrolled 933 patients with stroke in the first 24 hours of stroke onset, is one such example. It has been criticized because of the heterogeneous population included in the study, its slow recruitment rate, late treatment initiation, and inefficient glucose control. Seven other small studies did not have the statistical power to detect clinical efficiency.[13,15–20] They all showed that IIT carries a high risk of hypoglycemia (4%–76%). One of these studies[18] found that IIT was associated with greater infarct growth (IG) on MRI in patients with persistent arterial occlusion. However, the median delay of treatment initiation was 20 hours after stroke onset, so the study was unlikely to detect a putative protective effect on the ischemic penumbra. Additionally, better glucose control was not obtained in the intensive group, except in a small time window. To date, a larger study examining an intensive glycemic protocol versus usual care on the effects of glucose control and infarct expansion would be of great interest in patients with stroke. In such a study, it would be critical to start treatment as early as possible.[21]

The INSULINFARCT study was designed to address these issues. INSULINFARCT is a randomized proof-of-concept trial comparing IIT versus control subcutaneous treatment initiated within 6 hours of stroke onset in carotid infarction. The study was powered to detect an increased efficiency of IIT on serum glucose control during the next 24 hours compared with usual care with subcutaneous insulin. The working hypothesis was that IIT, by better glucose control, would reduce subsequent IG on MRI because hyperglycemia is independently associated with increased IG.[7,8,10,22]

Materials and Methods

Study Design

The INSULINFARCT study was an academic monocenter (Pitié-Salpêtrière Hospital, Paris, France), prospective, randomized, unblinded trial in patients with hyperacute stroke (<6 hours) to investigate insulin therapy management in the first 24 hours after admission. The first objective was to assess the efficiency of glycemic control achieved with intravenous insulin perfusion versus control subcutaneous insulin therapy over 24 hours. The second objective was to compare the MRI infarct growth between the 2 groups. The third objective was to compare the clinical and safety outcomes 90 days after stroke. The first and second objectives were planned to valid the working hypothesis, that is, that IIT, by better glucose control, would reduce subsequent IG on MRI. For recruitment reasons, the study initially designed for 2 years was extended to 4 years. There were no changes in the selection criteria or design during the recruitment period. The trial ended when the number of patients reached the required sample size for the primary objective.

Population

Eligible patients were prospectively enrolled and were consecutive patients from the Pitié-Salpêtrière Hospital admitted between June 2007 and March 2011 who met the following criteria: (1) an ischemic stroke in the carotid territory proven by initial diffusion-weighted imaging (DWI); (2) an initial MRI with DWI <5 hours of stroke onset; (3) initiation of insulin treatment within 1 hour of MRI (<6 hours after stroke onset); and (4) an admission score on the National Institutes of Health Stroke Scale (NIHSS) between 5 and 25. Patients were eligible whatever their baseline serum glucose level and their non- or diabetic status. Symptom onset was defined as the last time the patient was seen in normal health. The exclusion criteria were life-threatening conditions that limited follow-up visits, preadmission modified Rankin Scale >2, or patients under legal protection. The neurological examination was assessed using the NIHSS at admission (before the MRI and any treatment), on Days 1 and 7. Blood pressure, heart rate, and temperature were recorded at admission and on Day 1. Body mass index and umbilical perimeter were measured.

The study was conducted according to good clinical practice guidelines and was approved by the local ethical committee. Written informed consent was obtained by the on-duty physician from each participant or from a legal proxy/family member before randomization. Consent was not immediately required if the patient had severe language disturbances, neglect, or loss of consciousness so that the insulin treatment could be initiated as soon as possible.

Randomization and Treatment Protocol

Randomization and Masking

Each participant was randomized 1:1 by a secure web site (Cleanweb, Telemedicine Technologies) between 2 groups: the IIT (intravenous insulin continuously) or the SIT (subcutaneous insulin every 4 hours) group. Randomization was previously entered into the system using a prespecified randomization list (random-number generator). The randomization list was made of 4 blocks of 45 patients. None of the investigators were aware of the randomization list or the number or size of the blocks. Treatment allocation was performed during the 24 hours after randomization. Physicians were unblinded to the patients' treatment.

Treatment Protocol

In the IIT group, soluble human Actrapid insulin was administered in an intravenous continuous infusion with hourly dose adaptation to the capillary glucose test control (CGT) according to the nomogram shown in Figure 1A. In the SIT group, insulin was administered subcutaneously every 4 hours based on CGT values (Figure 1B). Nomograms were designed by the endocrinologic department. Insulin was stopped when the CGT value reached the lowest limit of the nomogram in each group. The stop point was <5.5 mmol/L in the IIT group and 8 mmol/L in the SIT group. Both groups have different target glucose thresholds because the aim of the study was to compare our usual management of glucose control (SIT group) with aggressive insulin therapy (IIT). Nevertheless, both nomograms were designed to achieve a targeted glucose control under the "toxic" threshold of 7 mmol/L. In both groups, the patients received saline infusion with potassium. Oral feeding was allowed if the neurological deficit was minor.

In case of hypoglycemia (defined as a CGT <3 mmol/L), the patient received 10 mL of 30% glucose infusion and CGT was checked every 15 minutes. If CGT reached the objective of the arm treatment, the doses of the allocated treatment were divided by 2.

After the 24 hours of the study treatment, the treatment was decided by the on-duty physician. The recommendation was to use

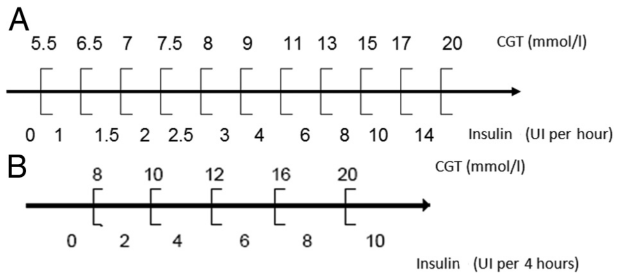

Figure 1. Intensive treatment nomogram by intravenous continuous insulin (**A**) and subcutaneous insulin (**B**).

Downloaded from http://stroke.ahajournals.org/ by guest on March 18, 2013

either subcutaneous insulin every 4 or 8 hours, antidiabetic drugs, or no treatment at all. Intravenous insulin infusion was recommended to be stopped.

MRI Data and Analysis

MRI Parameters

The initial MRI was performed at admission (<5 hours) before randomization and the control MRI was performed between Days 1 and 3.

MRI was performed on a 3.0-T whole-body MR unit (Signa 3.0-T HDx; General Electric Medical System, Milwaukee, WI) with an 8-channel head coil. The MRI protocol included 4 sequences: DWI, fluid-attenuated inversion recovery, intracranial time-of-flight MR angiography, and T2*-weighted sequence. Parameters could be found in Appendix I in the online-only Data Supplement.

MRI Assessment

The admission infarct volume was defined as the hyperintense area on initial DWI (b value=1000 s/mm^2). It was delineated by interactive manual outlining using the Neurinfarct software (Intelligence in Medical Technology, Paris, France). The follow-up infarct volume was determined using identical methodology applied to the follow-up DWI. This method has been shown to be highly reproducible.[23,24] The IG was defined as the difference between follow-up and admission DWI volumes. All volume determinations were performed by one author (C.R.) who was blinded to arm treatment.

Intracranial occlusion and arterial recanalization were assessed on initial and follow-up MR angiography, respectively. Recanalization was considered in a 3-item scale: (1) patent or complete recanalization; (2) partial or minimal flow-related signal in the region of the arterial clot or displacement of the initial clot in a distal portion of the occluded artery; and (3) persistent occlusion.

Blood Sample Collection

Patients had venous blood glucose measurement taken at admission before MRI and at the end of the treatment. Blood samples were obtained on Day 1 to detect diabetes mellitus and low-density lipoprotein cholesterol level. Blood glucose, diabetes mellitus, and low-density lipoprotein cholesterol measurements were centralized at the Pitié-Salpêtrière Hospital biochemical laboratory. CGTs were performed using an Optium H apparatus according to manufacturer procedures (ABBOTT-MediSense; www.abbottdiabetescare.co.uk/healthcare-professionals/clinical-papers).

Outcome Scales

The modified Rankin Scale (mRS) was used to assess the outcome at discharge and at 90 days. A good outcome was defined as independency (mRS 0–2) and a poor outcome as moderate to severe disability (mRS 3–5). This scale was performed by trained neurologists who were not blinded to the arm treatment.

Statistical Analysis

The analysis was performed as intention-to-treat to reflect the clinical practice in a stroke unit where patients with acute stroke are treated as soon as possible. Analysis was performed by the statistician of the trial (J.-C.C), blinded for treatment allocation, with Statistica software (StatSoft, Inc, Maison-Alfort, France).

Descriptive statistics are median and interquartile range (IQR). Comparisons of proportions were made by a χ^2 test; the quantitative variables were compared by *t* tests or repeated-measure analysis of variance. MRI volumes were compared by Mann-Whitney *U* tests because their distribution is not normal.[23,24] ORs and their 95% CI were computed for all binarized data as a measure of the effect size.

Glucose Control

The primary objective of the study was to test the hypothesis that the percentage of patients with a mean CGT <7 mmol/L during the 24-hour protocol would be higher in the IIT than in the SIT group. This was a necessary step to prove that intensive management of stroke-related hyperglycemia would modify infarct growth.

The mean CGT was defined as the averaged of CGT values determined at H4 (4 hours after randomization), H8, H12, H16, H20, and H24. We calculated that it was necessary to include 82 patients per group to detect a difference of 20% in the proportion of patients with a mean CGT <7 mmol/L with an α risk of 5% and a 90% power. Considering possible exclusions due to missing data, the sample size of both groups was fixed at 90. It was prespecified in the protocol that patients with >3 missing CGT values would be excluded. We also compared the CGT values in both groups at specific time points (H4, H8, H12, H16, H20, and H24), the proportion of patients with CGT <7 mmol/L at each of these time points.

Secondary Objectives

The subsequent prespecified secondary efficacy outcome variable was the comparison of IG on MRI. Based on previous data,[10,24] the study was sufficiently powered to detect a difference of 15 cm^3 in the infarct growth volume assuming a SD of 30 cm^3: 70 patients per group were necessary with an α risk of 5% and a 80% power. In addition, although this was not prespecified by the protocol, we had searched for treatment group and vessel patency effects on IG using a 2-way analysis of variance as was performed in a previous study.[18] We also performed a broad analysis of determinants of infarct growth by running a stepwise multiple regression analysis to adjust with the baseline variables. The IG was the dependent variable (as a continuous variable) and the independent variables were the baseline clinically relevant variables such as: age, arm of insulin treatment (SIT=1 and IIT=2), baseline NIHSS, diabetes mellitus binary status (≥6.2%), thrombolytic treatment, time between stroke and MRI, hypoglycemic events, and DWI volume at admission.

The other efficacy outcome, more exploratory, was the comparison of proportions of patients with good functional outcome at 3 months. We also analyzed functional outcome across the entire distribution of the mRS scores at Day 90 (χ^2 test, dl=6). We finally ran a logistic regression analysis with good functional outcome as the dependent variable and with the same independent variables as the previous regression analysis on IG. In addition, we compared NIHSS at Day 1 and Day 7 after stroke onset.

The prespecified safety outcomes were hypoglycemias (defined by CGT <3 mmol/L), death within 3 months, and serious adverse events including symptomatic intracerebral hemorrhages (as defined as any increase in NIHSS >3 points during the first week attributed to CT- or MRI-documented parenchymal hematomas), any neurological worsening (NIHSS >3 points), and any event that lengthened the hospital stay or was life-threatening.

Results

Population

Between June 2007 and March 2011, 230 patients were eligible and 180 were included in the study (Figure 2). The functional outcome and safety analyses were performed on the 180 patients in the intention-to-treat analysis. The analysis of glucose control was performed on 176 patients (89 in the SIT and 87 in the IIT groups) because 4 patients with >3 missing CGT values were excluded as prespecified in the protocol. In the per-protocol analysis, 6 patients were excluded because of violation of inclusion/exclusion criteria: vertebrobasilar stroke (n=2), mimicked stroke related to a dural fistula (n=1), preadmission mRS >2 (n=1), or because they were under legal protection (n=2). The MRI analysis was performed in 160 patients (80 in the control and 80 in the intensive group) because the control MRI was missing in 16 patients (6 early deaths, 3 comas, 7 follow-up MRIs performed after 3 days). Appendix II in the online-only Data

Downloaded from http://stroke.ahajournals.org/ by guest on March 18, 2013

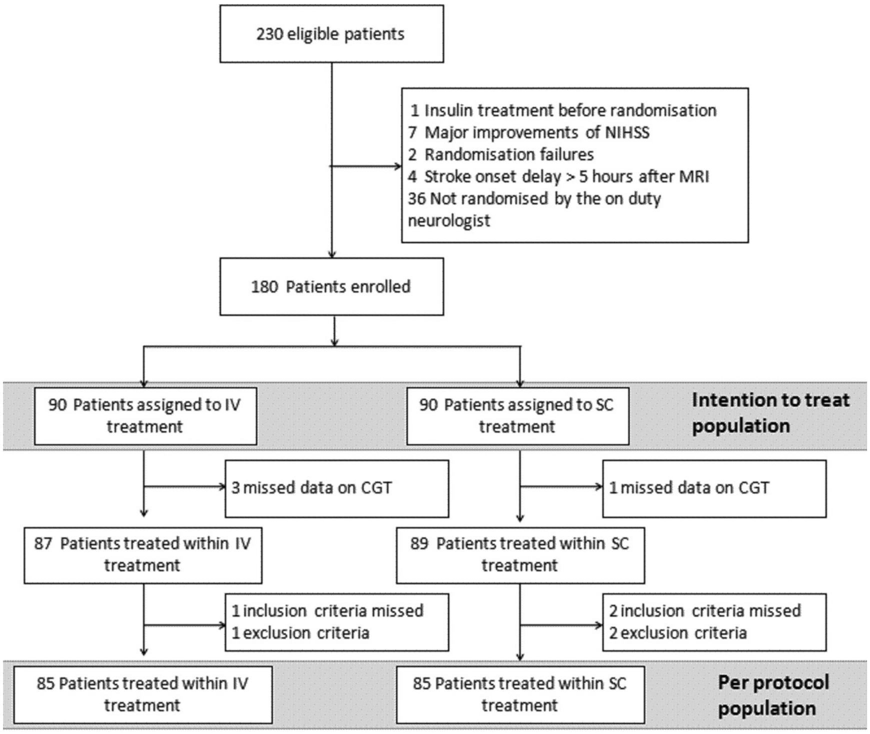

Figure 2. Flowchart of the study according to the CONSORT (CONsolidated Standards of Reporting Trials) guidelines. CGT indicates capillary glucose test; SC, subcutaneous; IV, intravenous.

Supplement provides details and sensitivity analysis on patients with missing MRI data in each group.

Patient characteristics are presented in Table 1. The body mass index was higher in the IIT group (P=0.02). Time to initial MRI tends to be earlier in the IIT group (P=0.06). Although there was no statistical difference between groups for the other variables, patients tend to be older and less frequently treated by recombinant tissue-type plasminogen activator in the SIT than in the IIT group. Fasting patients were 71% (63 of 89) in the SIT and 70% (61 of 87) in the IIT group. The number of patients under oral hypoglycemic agents before admission was 12.5% (13 of 89) in the SIT and 7% (6 of 87) in the IIT group. The initial CGTs were similar in both groups with a median at 6.6 mmol/L in the SIT group versus 6.7 mmol/L in the IIT group (P=0.48). Proportion of patients with initial CGT ≥7 mmol/L were 44% (39 of 89) in the SIT group versus 39% (34 of 87) in the IIT group (P=0.6). The median delay between initial MRI and randomization was similar (39 minutes; IQR, 32–49 versus 37 minutes; IQR, 29–48; P=0.89).

Glucose Control

The primary end point of a mean CGT <7 mmol/L was reached in 95.4% (83 of 87) of patients in the IIT and in 67.4% (60 of 89) of patients in SIT group (P<0.0001). The OR for the IIT group was 9.5 (95% CI, 3.2–28.6). The mean 24-hour CGT was lower in the IIT group (5.7 mmol/L; IQR, 5.2–5.9 versus 6.6 mmol/L; IQR, 5.6–7.3; P<0.0001). Figure 3 shows the CGT time course in both groups. The time course of CGT showed a significant group*time interaction (Figure 3; repeated-measure analysis of variance, P=0.005). The proportion of patients with a CGT <7 mmol/L at H4 was 86% in the IIT group and 58% in the SIT group (P=0.001; Figure 3). Nearly all patients (98%) received some insulin in the intensive group (24-hour dose, 18.5 UI; IQR, 9–24), whereas only 55% of patients received insulin in the control group (dose, 2 UI; IQR, 0–4; P<0.0001 between groups).

MRI Outcome

As shown in Table 1, the initial infarct volume was similar in both groups as was the percentage of intracranial arterial occlusions. The control MRI was performed at a similar delay in the IIT and SIT groups (29.5 hours; IQR, 27.2–36.2 versus 29.4 hours; IQR, 26.1–34.3; P=0.74). The IG was significantly larger in the IIT group (median, 27.9 cm^3; IQR, 3.5–64.2 versus 10.8 cm^3; IQR, 2.6–41; 60% of difference; P=0.04). The rate of middle cerebral artery recanalization was similar in the IIT and SIT groups (73.5% versus 79%; P=0.51). The analysis of IG according to intracranial arterial patency and treatment group (Figure 4) showed significant patency (F[2, 148]=10.7, P<0.0001) and treatment (F[1, 148]=4.4, P=0.03) effects but no significant interaction (F[2, 148]=2.1, P=0.12), although a larger IG occurred in patients with persistent arterial occlusion treated with intravenous insulin, like in McCormick et at.[18] The final model predicting the infarct growth (R^2=0.306, P<0.001) retained 3 variables in the model: arm of insulin treatment (coefficient 18.4, P=0.006), baseline NIHSS (coefficient: 2.6, P<0.0001), and admission DWI volume (coefficient: 0.4, P<0.0001). Variables not retained in the model were age, thrombolytic treatment, time between stroke onset and MRI, diabetes mellitus status, and hypoglycemic events.

Functional Outcome

Good functional outcome was achieved in the same proportion of patients in both groups (45.6% [41 of 90] versus 45.6% [41 of 90]; P=0.88) with an OR for the IIT group of 1 (95% CI, 0.6–1.8). The final model predicting good

Downloaded from http://stroke.ahajournals.org/ by guest on March 18, 2013

Table 1. Baseline Characteristics at Admission

	SIT (N=89)	IIT (N=87)
Age, y	76.9	69.6
	56.2–83.7	56.4–80.6
Sex, male/female, no. (%)	46 (51.7%)	50 (57.4%)
NIHSS at baseline	14	15
	8–20	8–19
Thrombolytics		
No. (%)	69 (77.5%)	75 (86.2%)
Time between stroke onset and tPA, min	173 (138–210)	164 (129–206)
Admission serum glucose level, mmol/L	6.3	6.7
	5.5–7.6	6.1–7.8
HBA1C, %	6	5.9
	5.6–6.1	5.4–6
Previously known diabetes, no. (%)	15 (16.8%)	7 (8%)
No. of patients with HBA1C ≥6.2%, no. (%)	22 (24.7%)	16 (18.4%)
LDL cholesterol, g/L	1.1 (0.9–1.4)	1 (0.8–1.5)
BMI*	25.5	26.7
	22.6–27.8	23.4–29.5
Umbilical perimeter, cm	95	97
	86–104	87–105
Systolic BP, mm Hg	145	151
	133–170	133–166
Diastolic BP, mm Hg	89	86
	79–99	77–99
Time to initial MRI, min	157	132
	110–202	99–182
Initial DWI volume, cm^3	10.5	11.4
	3.9–37.5	2.8–34.7
Intracranial occlusion		
No. (%)	71 (80%)	72 (83%)
Types of occlusions		
Branch of MCA (M2)	16 (22.5%)	15 (21%)
Trunk of MCA (M1)	33 (46.5%)	28 (39%)
ICA/MCA	10 (14%)	16 (22%)
T carotid occlusion	12 (17%)	13 (18%)

Numbers are median and interquartile range.

SIT indicates subcutaneous insulin therapy; IIT, intensive insulin therapy; NIHSS, National Institutes of Health Stroke Scale; tPA, tissue-type plasminogen activator; HBA1C, diabetes mellitus; LDL, low-density lipoprotein; BMI, body mass index; BP, blood pressure; DWI, diffusion-weighted imaging; MCA, middle cerebral artery; ICA, internal carotid artery.

*$P<0.05$ for between-group comparison.

functional outcome retained 3 variables: age (coefficient: −0.04, $P=0.0007$), baseline NIHSS (coefficient: −0.13, $P=0.0001$), and admission DWI volume (coefficient: −0.02, $P=0.01$). The distribution analysis across all scores of the mRS did not show any significant shift in the IIT group compared with the SIT group ($P=0.16$). NIHSS scores were similar between groups at Day 1 (15; IQR, 8–19 versus 14; IQR, 9–20; $P=0.63$) and Day 7 (11; IQR, 5–17 versus 10.5; IQR, 4–19; $P=0.63$).

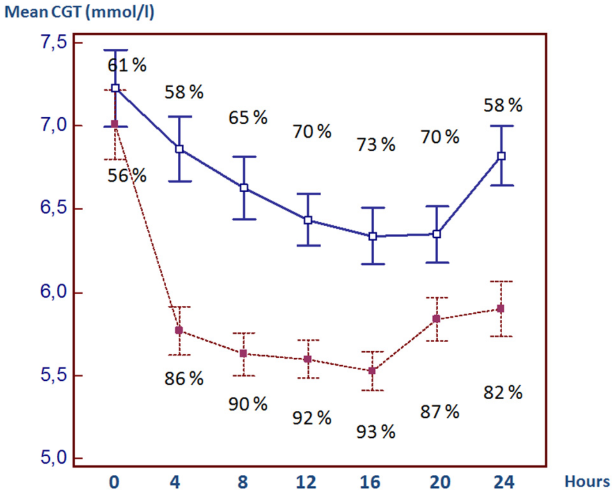

Figure 3. Impact of treatment group on glycemic control. Mean capillary blood glucose test at admission, H4, H8, H12, H16, H20, and H24 after treatment allocation in the IIT and the SIT groups. Proportions of patients with CGT <7 mmol/L are indicated on the graph at each time point and they were all significantly different. The repeated-measure ANOVA showed a significant treatment group effect ($F[1, 163]=19.8$, $P<0.0001$) with a significant time effect ($F=21.8$, $P<0.0001$) and a significant group*time interaction ($F[6, 978]=3.1$, $P=0.005$). H indicates hour; IIT, intensive insulin therapy; SIT, subcutaneous insulin therapy; CGT, capillary glucose test; ANOVA, analysis of variance.

Safety Outcome

Five patients (5.7%) had 8 asymptomatic hypoglycemic episodes in the IIT group versus none in the SIT group ($P=0.07$ for the number of patients and $P=0.02$ for the number of events). None of the hypoglycemic episodes was considered symptomatic (worsening in NIHSS or consciousness disturbance). If we consider a less conservative threshold to define hypoglycemia (≤3.6 mmol/L), these rates were 1.1% (one of 89) versus 34.5% (30 of 87) of hypoglycemic events for 1.1% (one of 89) and 19.5% (17 of 87) patients in the SIT versus the IIT group.

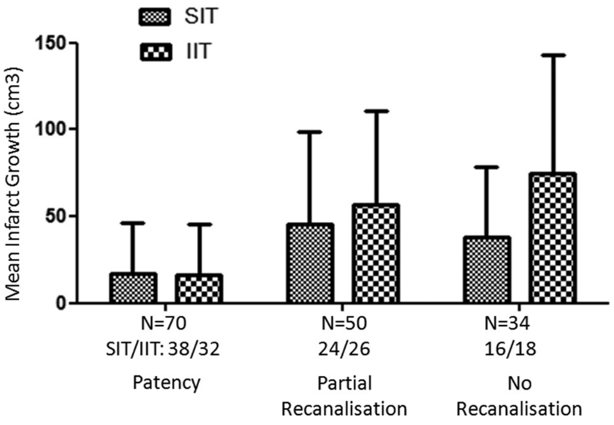

Figure 4. Effect of treatment group and arterial recanalization on infarct growth. Histograms of mean infarct growth in the SIT and IIT group for each recanalization status: patent, partial recanalization, or persistent occlusion. Error bars represent the SEM. SIT indicates subcutaneous insulin therapy; IIT, intensive insulin therapy.

Downloaded from http://stroke.ahajournals.org/ by guest on March 18, 2013

Table 2. Serious Adverse Events (SAEs) During the Trial

	SIT (N=90)	IIT (N=90)
Death, no. (%)	14 (15.6%)	9 (10%)
Patients with SAE, no. (%)	32 (35.6%)	25 (27.7%)
SAE including death, no. (%)	49 (54.4%)	41 (45.6%)
SAE, no. (%)	35 (38.9%)	32 (35.6%)
Neurological causes	23/35 (66%)	25/32 (78.5%)
Extension/edema	13 (37%)	8 (25%)
Symptomatic IC hemorrhage	6 (17%)	7 (22%)
Symptomatic EC hemorrhage	1 (3%)	4 (12.5%)
New events (TIA, stroke)	1 (3%)	3 (9.5%)
Epilepsy	2 (6%)	3 (9.5%)
Extraneurological causes	12/35 (34%)	7/32 (21.5%)
Pulmonary infection	5 (14%)	1 (3.1%)
Pulmonary embolism	1 (3%)	1 (3.1%)
Pulmonary edema	3 (9%)	3 (9.2%)
Coronary events	1 (3%)	2 (6.1%)
Others	2 (5%)	0 (0%)

P values for between-group comparisons were >0.05.

SIT indicates subcutaneous insulin therapy; IIT, intensive insulin therapy; IC, intracranial; EC, extracranial; TIA, transient ischemic attack.

Analysis of safety data found 32 versus 25 patients with serious adverse events in the SIT and IIT groups, respectively (Table 2; $P=0.33$). There was no significant difference in death at 3 months in the 2 groups ($P=0.36$) with an OR for the IIT group of 0.6 (95% CI, 0.25–1.5). Serious adverse events including or not including death occurred in the same proportions in the 2 treatment arms. The most frequent serious adverse event was neurological worsening due to infarct extension or malignant edema. Symptomatic intracranial hemorrhage occurred in 7 patients in the IIT group and 6 in the SIT group. Asymptomatic intracranial hemorrhages, with no neurological worsening, were similar in the SIT and IIT groups (34 of 88 [38%] versus 35 of 89 [39%]; $P=0.98$).

Discussion

This trial has confirmed a better control of CGT by an intensive intravenous treatment with insulin versus a usual subcutaneous insulin protocol. The median delay of treatment initiation was <3 hours and efficient glucose control was quickly achieved in the IIT group. The IIT treatment was more effective than the SIT treatment in controlling capillary glucose level early after stroke with 95.4% of patients having a mean CGT <7 mmol/L during the 24 hours of treatment. Furthermore, efficient glucose control was observed in 86% of the patients at the first time point, which was 4 hours after the initiation of treatment. In addition, the intravenous saline–insulin regimen appeared to be safe despite more hypoglycemic episodes but not associated with more serious adverse events. Hypoglycemia occurred only in 5.7% (n=5) of the IIT group, a rate in the lower range of previously reported stroke IIT studies,[13,14,16,18] showing that this saline infusion insulin nomogram was at least as safe as glucose–potassium–insulin regimens previously reported.[14] In addition, the study was performed in an intensive care unit with highly trained nurses, previously formed to other protocols using scaled therapies. However, hypoglycemic events were not retained in the logistic regression analysis predicting good functional outcome. There were no hypoglycemic episodes in the SIT group.

Despite the excellent efficiency–safety profile of the IIT regimen, the infarct growth was not smaller in the IIT group. This might be disappointing because animal[5,25] and human[6–9] imaging data have shown that acute hyperglycemia increases infarct growth by exaggerating the transformation of penumbra into infarction and perhaps reperfusion injury. However, our results are consistent with those of McCormick and colleagues[18] who reported that IIT was not associated with reduced infarct growth but with an increased IG in patients with persistent occlusion in a small series of patients. In the present study, the largest infarct growth was also observed in the IIT persistent occlusion group. Furthermore, several studies have shown that preischemic insulin reduces IG in hyperglycemic animals, but there are very few reports on the effects of postischemic insulin treatment.[5,26,27] In one study[27] involving 12 rats, early death occurred in 5 animals and infarct size in the remaining rats was similar to those of the control groups. Similarly, 4 of 10 hyperglycemic cats receiving postinfarct insulin died from malignant edema and the remaining had the largest infarct size.[26] We have no explanation for this discrepancy between preclinical and clinical data but they clearly indicate the need for a reappraisal of the pathophysiological models of glucose energy metabolism alterations in the early phase of focal cerebral ischemia.

Despite the increased infarct growth in the IIT group, the functional outcome was similar in both groups perhaps because the study was not powered to detect clinical changes.

This study has some limitations due to its monocentric and open-label design. However, the patient sample was representative of the usual care of the population treated in our stroke center because 78% of eligible patients were included in the study. Although DWI was found highly correlated with relevant clinical outcome in previous studies, DWI volumetry only allows an approximation of initial and final infarct size.[10,28] Nevertheless, follow-up DWI volume performed between Day 1 and 3 could be also an advantage because it decreases lost at follow-up and dead at follow-up patients and it has been shown to be highly correlated with 30-days fluid-attenuated inversion recovery-determined volume.[29] The study was probably underpowered to detect a modest clinical change on the functional outcome although a favorable functional outcome in favor of IIT seems unlikely when considering the negative result found on DWI. Another and independent limit concerns that only few patients included in this study were diabetic (18% and 24%, respectively, in the IIT and SIT groups) and even few have very high glucose levels. This may limit the conclusions for this specific population for whom the poststroke hyperglycemia profile might be managed differently.[30]

Summary

In conclusion, in the INSULINFARCT study, the working hypothesis was not confirmed; despite rapid and efficient

Downloaded from http://stroke.ahajournals.org/ by guest on March 18, 2013

control of poststroke hyperglycemia in acute stroke, intravenous insulin-controlled glucose did not reduce infarct growth but rather increased it. IIT cannot be recommended at the present time.

Acknowledgments

We thank all of the nursing staff who participated during this 4-year protocol.

Sources of Funding

INSULINFARCT was promoted by the Assistance Publique Hôpitaux de Paris (AP-HP). The AP-HP had no role in the design, data analysis, or report writing.

Disclosures

None.

References

1. Bruno A, Levine SR, Frankel MR, Brott TG, Lin Y, Tilley BC, et al. Admission glucose level and clinical outcomes in the NINDS rtPA stroke trial. *Neurology*. 2002;59:669–674.
2. Capes SE, Hunt D, Malmberg K, Pathak P, Gerstein HC. Stress hyperglycemia and prognosis of stroke in nondiabetic and diabetic patients: a systematic overview. *Stroke*. 2001;32:2426–2432.
3. Diener HC, Lees KR, Lyden P, Grotta J, Davalos A, Davis SM, et al. NXY-059 for the treatment of acute stroke: pooled analysis of the SAINT I and II trials. *Stroke*. 2008;39:1751–1758.
4. Kruyt ND, Biessels GJ, Devries JH, Roos YB. Hyperglycemia in acute ischemic stroke: pathophysiology and clinical management. *Nat Rev Neurol*. 2010;6:145–155.
5. MacDougall NJ, Muir KW. Hyperglycaemia and infarct size in animal models of middle cerebral artery occlusion: systematic review and meta-analysis. *J Cereb Blood Flow Metab*. 2011;31:807–818.
6. Alvarez-Sabin J, Molina CA, Ribo M, Arenillas JF, Montaner J, Huertas R, et al. Impact of admission hyperglycemia on stroke outcome after thrombolysis: risk stratification in relation to time to reperfusion. *Stroke*. 2004;35:2493–2498.
7. Baird TA, Parsons MW, Phanh T, Butcher KS, Desmond PM, Tress BM, et al. Persistent poststroke hyperglycemia is independently associated with infarct expansion and worse clinical outcome. *Stroke*. 2003;34:2208–2214.
8. Parsons MW, Barber PA, Desmond PM, Baird TA, Darby DG, Byrnes G, et al. Acute hyperglycemia adversely affects stroke outcome: a magnetic resonance imaging and spectroscopy study. *Ann Neurol*. 2002;52:20–28.
9. Ribo M, Molina CA, Delgado P, Rubiera M, Delgado-Mederos R, Rovira A, et al. Hyperglycemia during ischemia rapidly accelerates brain damage in stroke patients treated with tPA. *J Cereb Blood Flow Metab*. 2007;27:1616–1622.
10. Rosso C, Attal Y, Deltour S, Hevia-Montiel N, Lehericy S, Crozier S, et al. Hyperglycemia and the fate of apparent diffusion coefficient-defined ischemic penumbra. *AJNR Am J Neuroradiol*. 2011;32:852–856.
11. Garg R, Chaudhuri A, Munschauer F, Dandona P. Hyperglycemia, insulin, and acute ischemic stroke: a mechanistic justification for a trial of insulin infusion therapy. *Stroke*. 2006;37:267–273.
12. Quinn TJ, Lees KR. Hyperglycaemia in acute stroke—to treat or not to treat. *Cerebrovasc Dis*. 2009;27(suppl 1):148–155.
13. Bruno A, Kent TA, Coull BM, Shankar RR, Saha C, Becker KJ, et al. Treatment of Hyperglycemia in Ischemic Stroke (THIS): a randomized pilot trial. *Stroke*. 2008;39:384–389.
14. Gray CS, Hildreth AJ, Sandercock PA, O'Connell JE, Johnston DE, Cartlidge NE, et al. Glucose-potassium-insulin infusions in the management of post-stroke hyperglycaemia: the UK Glucose Insulin in Stroke Trial (GIST-UK). *Lancet Neurol*. 2007;6:397–406.
15. Hamilton MG, Tranmer BI, Auer RN. Insulin reduction of cerebral infarction due to transient focal ischemia. *J Neurosurg*. 1995;82:262–268.
16. Johnston KC, Hall CE, Kissela BM, Bleck TP, Conaway MR. Glucose Regulation in Acute Stroke Patients (GRASP) trial: a randomized pilot trial. *Stroke*. 2009;40:3804–3809.
17. Kreisel SH, Berschin UM, Hammes HP, Leweling H, Bertsch T, Hennerici MG, et al. Pragmatic management of hyperglycaemia in acute ischaemic stroke: safety and feasibility of intensive intravenous insulin treatment. *Cerebrovasc Dis*. 2009;27:167–175.
18. McCormick M, Hadley D, McLean JR, Macfarlane JA, Condon B, Muir KW. Randomized, controlled trial of insulin for acute poststroke hyperglycemia. *Ann Neurol*. 2010;67:570–578.
19. Staszewski J, Brodacki B, Kotowicz J, Stepien A. Intravenous insulin therapy in the maintenance of strict glycemic control in nondiabetic acute stroke patients with mild hyperglycemia. *J Stroke Cerebrovasc Dis*. 2010;20:150–154.
20. Walters MR, Weir CJ, Lees KR. A randomised, controlled pilot study to investigate the potential benefit of intervention with insulin in hyperglycaemic acute ischaemic stroke patients. *Cerebrovasc Dis*. 2006;22:116–122.
21. Johnston KC, Parsons M. Aggressive glucose control in acute stroke: is the answer in the imaging? *Ann Neurol*. 2010;67:557–558.
22. Piironen K, Putaala J, Rosso C, Samson Y. Glucose and acute stroke: evidence for an interlude. *Stroke*. 2012;43:898–902.
23. Luby M, Bykowski JL, Schellinger PD, Merino JG, Warach S. Intra- and interrater reliability of ischemic lesion volume measurements on diffusion-weighted, mean transit time and fluid-attenuated inversion recovery MRI. *Stroke*. 2006;37:2951–2956.
24. Rosso C, Hevia-Montiel N, Deltour S, Bardinet E, Dormont D, Crozier S, et al. Prediction of infarct growth based on apparent diffusion coefficients: penumbral assessment without intravenous contrast material. *Radiology*. 2009;250:184–192.
25. de Courten-Myers G, Myers RE, Schoolfield L. Hyperglycemia enlarges infarct size in cerebrovascular occlusion in cats. *Stroke*. 1988;19:623–630.
26. de Courten-Myers GM, Kleinholz M, Wagner KR, Myers RE. Normoglycemia (not hypoglycemia) optimizes outcome from middle cerebral artery occlusion. *J Cereb Blood Flow Metab*. 1994;14:227–236.
27. Zhu CZ, Auer RN. Optimal blood glucose levels while using insulin to minimize the size of infarction in focal cerebral ischemia. *J Neurosurg*. 2004;101:664–668.
28. Tourdias T, Renou P, Sibon I, Asselineau J, Bracoud L, Dumoulin M, et al. Final cerebral infarct volume is predictable by MR imaging at 1 week. *AJNR Am J Neuroradiol*. 2011;32:352–358.
29. Campbell BC, Tu HT, Christensen S, Desmond PM, Levi CR, Bladin CF, et al. Assessing response to stroke thrombolysis: validation of 24-hour multimodal magnetic resonance imaging. *Arch Neurol*. 2012;69:46–50.
30. Bruno A, Saha C, Williams LS, Shankar R. IV insulin during acute cerebral infarction in diabetic patients. *Neurology*. 2004;62:1441–1442.

Downloaded from http://stroke.ahajournals.org/ by guest on March 18, 2013

SUPPLEMENTAL MATERIAL

1. Appendix 1 : MRI parameters

Axial isotropic DWI spin echo EPI included 24 slices of 5 mm thickness, with an interslice gap of 0.5 mm, a 280x280 mm FOV, a 96x128 matrix, TR/TE= 5800/86.4 ms. A baseline T2 acquisition and a diffusion-weighted acquisition using a diffusion gradient of 1000 s/mm2 were both acquired within 62 seconds. Axial fast-FLAIR sequence parameters were: 5-mm axial with an interslice gap of 0.5 mm, 256x192 matrix, 240x240 mm FOV, TR/TE = 9800/159 ms, inversion time (TI) = 2300 ms. Time-of-flight MR angiography was set to the following parameters: vascular time-of-flight by spoiled gradient-recalled acquisition, 1.4-mm axial slice thickness, 256x192 matrix, 240x240 mm FOV, TR = 34 ms, effective TE = 6.3 ms, FA of 25° for an acquisition time of 2 minutes 39 seconds. The T2*-weighted sequence parameters were: 5-mm axial slices with an interslice gap of 0.5 mm, 256x128 matrix, 240x240 mm field-of-view (FOV), repetition time (TR) = 580 ms, echo time (TE) =15 ms, flip angle (FA)=20°.

Downloaded from http://stroke.ahajournals.org/ by guest on March 18, 2013

Appendix 2 : Analyses concerning patients with missing MRI data.

In this trial, we had 11% (20/180) of missing MRI data in the intention-to-treat analysis. Ten MRI data (5.5%) were missing in each arm of the treatment.

Here we report the comparison of infarct growth between the SIT (Subcutaneous Insulin Therapy) and the IIT (Intensive Insulin Therapy) concerning that missing patients for the secondary objective.

First, we have compared the baseline variables in these two groups of subjects (see the table below).

There was no statistical difference (p>0.05) for any of these baseline variables, showing that bias due to missing data was minimized.

Median, IQR	SIT<br>N=10	IIT<br>N=10
Age (years)	84<br>66-89	73<br>64-81
Sex (M/F) n (%)	4/6 (40%)	5/5 (50%)
NIHSS at baseline	18<br>8-24	21<br>11-23
Thrombolytics<br>- n, (%)<br>Time between stroke onset-tPA (min)	 7 (70%)<br>162	 7 (70%)<br>189
Admission serum glucose level (mmol/l)	7.5<br>6.5-8.2	6.9<br>6.1-8.5
Time to initial MRI (min)	150<br>108-208	147<br>122-206

Secondly, we have performed a sensitivity analysis on the whole population (n=180), with a missing data imputation for 20 patients. We have chosen to impute the missing infarct growth volumes by the median of the infarct growth which corresponds to the 3-months functional outcome of each patient.

The median of the infarct growth based on the 3-months modified Rankin Scale (mRS) in the whole population was :

Downloaded from http://stroke.ahajournals.org/ by guest on March 18, 2013

	Median infarct growth (cm^3)
3-months mRS 0 to 2	3.7
3-months mRS 3 to 5	42.6
3 months MRS 6	27.5

After missing data imputation, we computed a Mann Whitney U test to compare the infarct growth volume in the SIT and IIT groups.

The median IG was still lower in the SIT group than in the IIT group (median, 95%CI: 13.7, 7.5-27.4 cm^3 vs. 27.5, 16.6-38.4 cm^3, p=0.04).

Downloaded from http://stroke.ahajournals.org/ by guest on March 18, 2013
