# Supplementary material for: Hyperglycaemia, Insulin Therapy and Critical Penumbral Regions for Prognosis in Acute Stroke: Further Insights from the INSULINFARCT Trial
Source: PLoS One. 2015 Mar 20;10(3):e0120230. doi: 10.1371/journal.pone.0120230 (PMC4368038; doi:10.1371/journal.pone.0120230)
Supplement: S1 File — (DOC) [file pone.0120230.s003.doc]

**Supporting information S1_File**

MRI Parameters

Axial isotropic DWI spin echo EPI included 24 slices of 5 mm thickness, with an interslice gap of 0.5 mm, a 280x280 mm FOV, a 96x128 matrix, TR/TE= 5800/86.4 ms. A baseline T2 acquisition and a diffusion-weighted acquisition using a diffusion gradient of 1000 s/mm2 were both acquired within 62 seconds. Axial fast-FLAIR sequence parameters were: 5-mm axial with an interslice gap of 0.5 mm, 256x192 matrix, 240x240 mm FOV, TR/TE = 9800/159 ms, inversion time (TI) = 2300 ms. Time-of-flight MR angiography was set to the following parameters: vascular time-of-flight by spoiled gradient-recalled acquisition, 1.4-mm axial slice thickness,  256x192 matrix, 240x240 mm FOV, TR = 34 ms, effective TE = 6.3 ms, FA of 25° for an acquisition time of 2 minutes 39 seconds. The T2*-weighted sequence parameters were: 5-mm axial slices with an interslice gap of 0.5 mm, 256x128 matrix, 240x240 mm field-of-view (FOV), repetition time (TR) = 580 ms, echo time (TE) =15 ms, flip angle (FA)=20°.
