# Supplementary material for: Hyperglycaemia, Insulin Therapy and Critical Penumbral Regions for Prognosis in Acute Stroke: Further Insights from the INSULINFARCT Trial
Source: PLoS One. 2015 Mar 20;10(3):e0120230. doi: 10.1371/journal.pone.0120230 (PMC4368038; doi:10.1371/journal.pone.0120230)
Supplement: S2 File — (DOC) [file pone.0120230.s004.doc]

**Supporting information S2_File**

Diffusion Magnetic Resonance Imaging (dMRI) Analysis

For tractography, we used a set of diffusion imaging acquired in one subject of the Human Connectome Project (HCP) from the Q3 data release that was available since September 2013 (<http://www.humanconnectome.org/>). MR datasets of the HCP are collected from each participant during a visit to Washington University that includes a diffusion imaging sequence that we used here. The HCP subject was scanned on a customized Siemens 3T “Connectome Skyra” housed at Washington University in St. Louis, using a standard 32-channel Siemens receive head coil and a “body” transmission coil designed by Siemens specifically for the smaller space available using the special gradients of the WU-Minn and MGH-UCLA Connectome scanners (1). Acquisition parameters were: Spin echo EPI, 210x180 mm FOV, a 168x144 matrix, TR/TE= 5520/89.5 ms, isotropic voxel size: 1.25 mm3, b_value=2000 s/mm2. The full dMRI session includes 6 runs (each approximately 9 minutes and 50 seconds), representing 3 different gradient tables, with each table acquired once with right-to-left and left- to-right phase encoding polarities, respectively. Each gradient table includes approximately 90 diffusion weighting directions plus 6 b=0 acquisitions interspersed throughout each run. The diffusion directions were obtained using a toolbox available from INRIA that returns uniformly distributed directions in multiple q-space shells.

The HCP MRI data pre-processing pipelines are primarily built using tools from FSL (2, 3).The diffusion preprocessing pipeline does the following: normalizes the b0 image intensity across runs; removes EPI distortions, eddy-current-induced distortions, and subject motion; corrects for gradient-nonlinearities; brings it into 1.25mm structural space; and masks the data with the final brain mask.

Probabilistic distributions of the fiber orientations (4) were then calculated for each voxel using a constrained spherical deconvolution model using MRTRIX software (5, 6). Probabilistic tractography was performed with a region-of-interest (ROI) approach. The main ROI used was the intersection between the cluster generated by the voxel-based analysis on HG and NG patients and the cluster generated by the analysis comparing poor vs. good outcome group. Because this ROI was in the normalized space (MNI), it was coregistered to fit the « connectome » subject’s DTI space (SPM8, VBM8 toolbox), and was rendered binary. Projections of the tracts that passed through this ROI were described to understand the anatomical connectivity of this region using Trackvis (version 0.4.2, Boston, USA).

References

1. Van Essen DC, Smith SM, Barch DM, Behrens T, Yacoub E, Ugurbil K, for the WU-Minn HCP Consortium. The WU-Minn Human Connectome Project: An overview. *NeuroImage.* 2013;80:62-79
2. Glasser MF, Sotiropoulos SN, Wilson JA, Coalson TS, et al. The minimal preprocessing pipelines for the Human Connectome Project. *Neuroimage.* 2013;80: 105-124.
3. Jenkinson M, Beckmann CF, Behrens TE, Woolrich MW, Smith SM. FSL. *NeuroImage.* 2012; 62:782-790.
4. Behrens TE, Berg HJ, Jbabdi S, Rushworth MF, Woolrich MW. Probabilistic diffusion tractography with multiple fibre orientations: What can we gain? *Neuroimage.* 2007;34:144-155
5. Tournier JD, Calamante F, Gadian DG, Connelly A. Direct estimation of the fiber orientation density function from diffusion-weighted MRI data using spherical deconvolution*. Neuroimage.* 2004;23:1176-1185
6. Tournier JD, Calamante F, Connelly A. Robust determination of the fibre orientation distribution in diffusion MRI: Non-negativity constrained super-resolved spherical deconvolution. *Neuroimage.* 2007;35:1459-1472

Acknowledgments section

Data were provided [in part] by the Human Connectome Project, WU-Minn Consortium (Principal Investigators: David Van Essen and Kamil Ugurbil; 1U54MH091657) funded by the 16 NIH Institutes and Centers that support the NIH Blueprint for Neuroscience Research; and by the McDonnell Center for Systems Neuroscience at Washington University
