# Supplementary material for: Hyperglycaemia, Insulin Therapy and Critical Penumbral Regions for Prognosis in Acute Stroke: Further Insights from the INSULINFARCT Trial
Source: PLoS One. 2015 Mar 20;10(3):e0120230. doi: 10.1371/journal.pone.0120230 (PMC4368038; doi:10.1371/journal.pone.0120230)
Supplement: S1 Table — (DOC) [file pone.0120230.s005.doc]

**Supporting S1 Table.** Characteristics of the intention-to-treat population in the INSULINFARCT trial, of the included and excluded population for this study.

| Median  IQR | INSULINFARCT  N=180 | Included group  N=99 | Excluded group  N=81 |
| --- | --- | --- | --- |
| Age (years) | 73  56-82 | 71  56-83 | 74  58-83 |
| Sex (male) n, % | 35  55% | 57  57% | 43  53% |
| Baseline NIHSS | 15  9-20 | 13  8-19 | 16  9-21 |
| rtPA use n, % | 145  81% | 79  80% | 66  81% |
| Time to MRI (minutes) | 141  102-190 | 134  100-180 | 155  108-199 |
| CGT before treatment  (mmol/l) | 6.6  5.8-8.1 | 6.7  5.8-8.1 | 6.6  5.8-8.2 |
| Insulin Regimen (IIT)  N, % | 90  50% | 53  53% | 37  46% |
| Patients with admission CGT < 7 mmol/l (n, %) | 101/178  57% | 54/97  56% | 27/80  34% |
| 24-hours mean CGT  (mmol/l)* | 5.9  5.3-6.7 | 5.8  5.3-6.6 | 6  5.4-6.8 |
| Patients with 24-hours mean CGT <7 mmol/l (n, %) | 144  82% | 84  84% | 60  77% |
| Recanalisation (n, %) | 127/161  79% | 77/97  79% | 50/64  78% |
| Day seven NIHSS | 6  2-15 | 5  1-12 | 6  3-19 |
| mRS 02 (n, %) | 81/177  46% | 54/97  56% | 27/80  34% |
| Initial volume (cm^3^) | 10.5  3.5-35.4 | 8.8  2.6-26.6 | 19.6  3.9-48.8 |
| Day one volume (cm^3^)* | 30.3  7-97.5 | 23.9  5.8-72 | 57.6  10.4-126-5 |
| Infarct growth (cm^3^) | 17.2  2.7-49.2 | 14.1  2 .1-38.9 | 31.6  3.8-84.2 |

CGT means Capillary glucose Test ; mRS : modified Rankin scale ; IIT : Intensive Insulin Treatment, * means p < 0.05 between the included and excluded subgroups.
