# Supplementary figures and images for: Hyperglycaemia, Insulin Therapy and Critical Penumbral Regions for Prognosis in Acute Stroke: Further Insights from the INSULINFARCT Trial
Source: PLoS One. 2015 Mar 20;10(3):e0120230. doi: 10.1371/journal.pone.0120230 (PMC4368038; doi:10.1371/journal.pone.0120230)

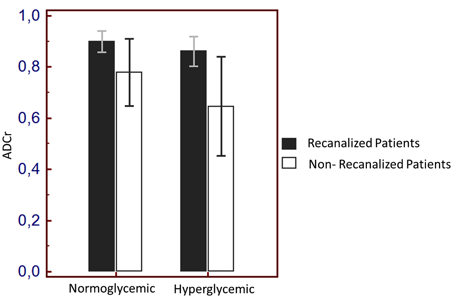

Supplement: S1 Fig — Dark grey represents recanalized patients and white bars non-recanalized patients. Bars represent mean; error bars represent 95% confidence interval of the mean. (TIF) [file pone.0120230.s006.tif]
